# Supplementary figures and images for: Effects of therapeutic vaccination on the control of SIV in rhesus macaques with variable responsiveness to antiretroviral drugs
Source: PLoS One. 2021 Jun 17;16(6):e0253265. doi: 10.1371/journal.pone.0253265 (PMC8211199; doi:10.1371/journal.pone.0253265)

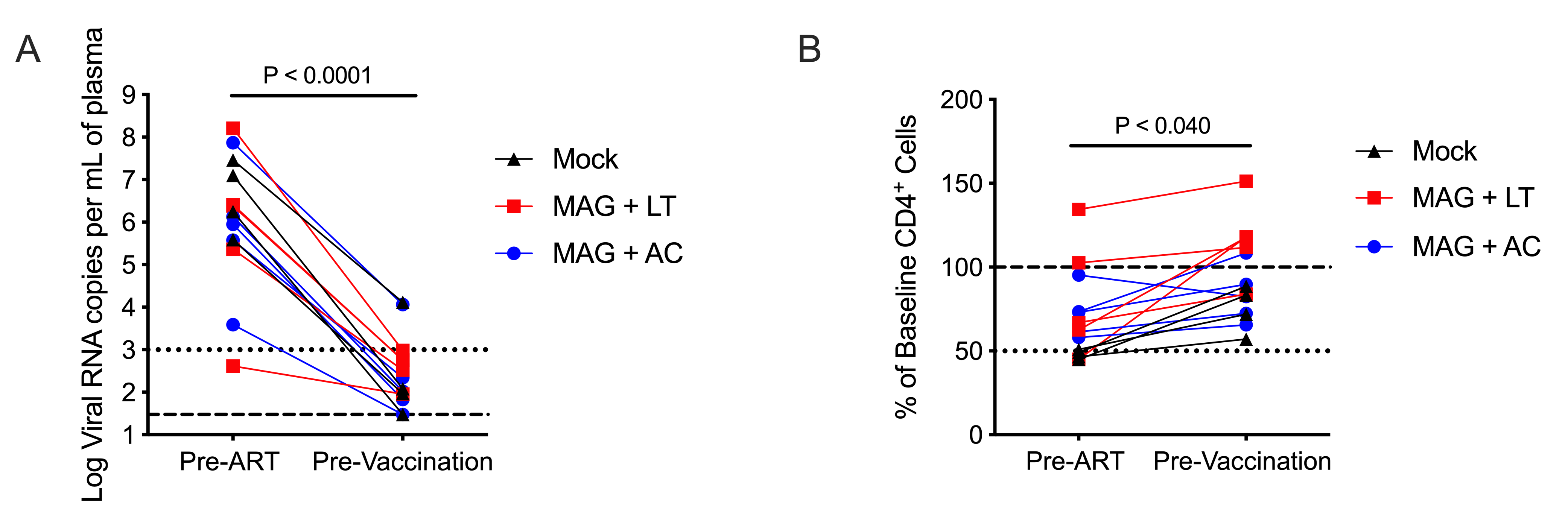

Supplement: S1 Fig — (A) Plasma viral loads were determined by RT-q-PCR for the mock (black circles), MAG + LT (red squares) and MAG + AC (purple triangles) groups. The dashed line indicates the assay limit of detection (30 viral RNA copies/1mL of plasma) and the dotted line indicates the threshold for control of virus replication. Shown is the decrease of each animals’ viral loads between pre-ART (6 wpi) and pre-vaccination (32 wpi). Statistical analyses were performed using a Wilcoxon matched-pairs signed rank test; results are considered significant if P ≤ 0.05. (B) Percent of baseline CD4+ T cell counts were calculated for the mock, MAG + LT and MAG + AC groups over time by dividing the absolute CD4+ count at a timepoint by the absolute CD4+ count at 0 wpi and multiplying by 100. The dotted line indicates 50% of baseline CD4+ T cells. CD4 T cell counts were obtained using a Beckman Coulter® AC*T™ 5diff hematology analyzer. Shown is the restoration of each animals’ percent of baseline CD4+ T cell counts between pre-ART (6 wpi) and pre-vaccination (32 wpi). Statistical analyses were performed using a Wilcoxon matched-pairs signed rank test; results are considered significant if P ≤ 0.05. (TIF) [file pone.0253265.s001.tif]

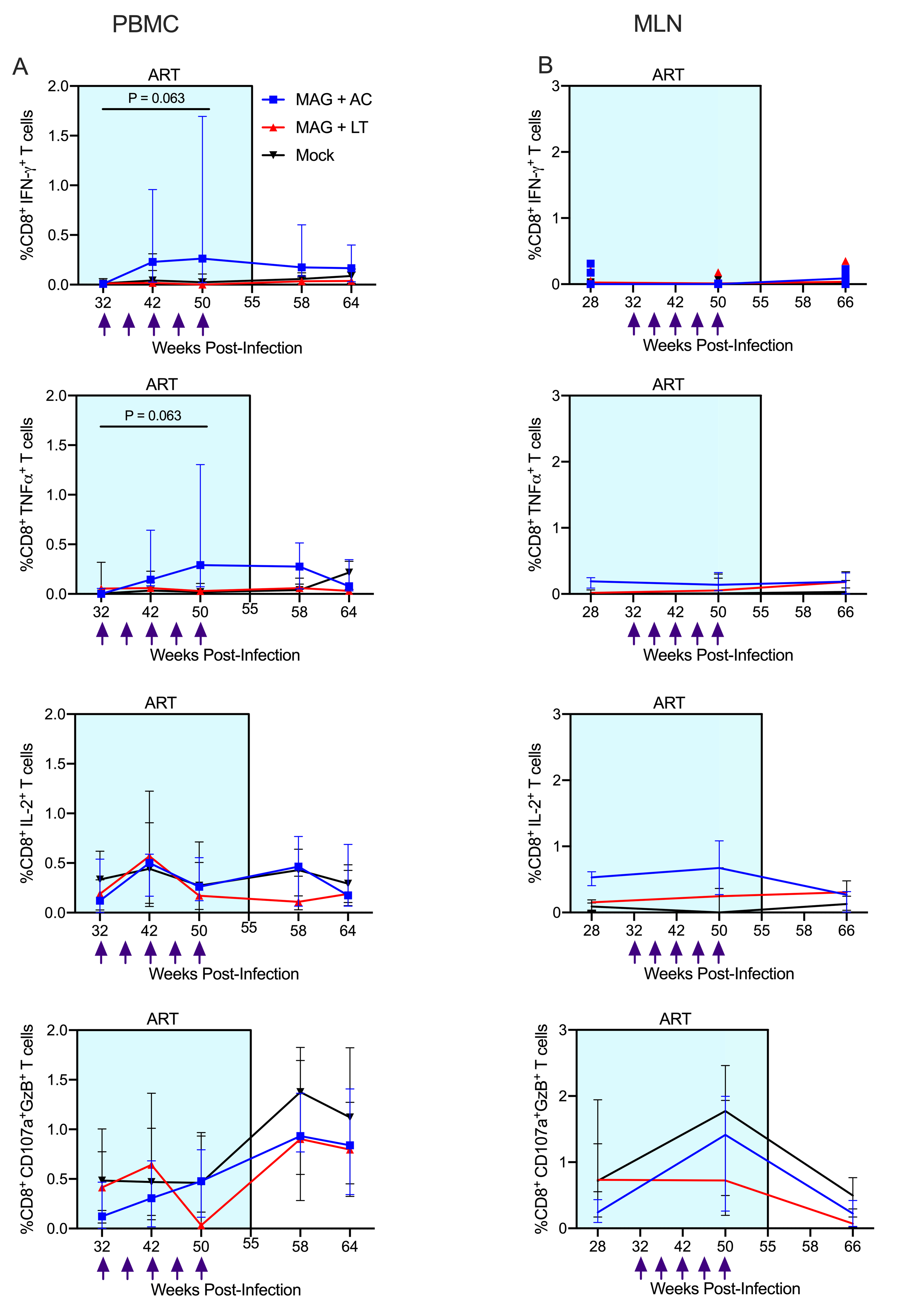

Supplement: S2 Fig — (A) PBMCs were thawed and stimulated with Gag peptides, and expression of IL-2, IFN-γ, TNFα and CD107a/GzB were quantified using intracellular cytokine staining. Shown are the medians and interquartile ranges of each group’s SIV-Gag specific T cell response. (B) Lymphocytes isolated from MLN were thawed and stimulated with Gag peptides, and expression of IL-2, IFN-γ, TNFα and CD107a/GzB were quantified using intracellular cytokine staining. Shown are the medians and interquartile ranges of each group’s SIV-Gag specific T cell response. (A, B) Statistics. Statistical comparisons between baseline and post-vaccination timepoints within a group were calculated using a Wilcoxon matched-pairs signed rank test. Results are considered significant if P ≤ 0.05. (TIF) [file pone.0253265.s002.tif]

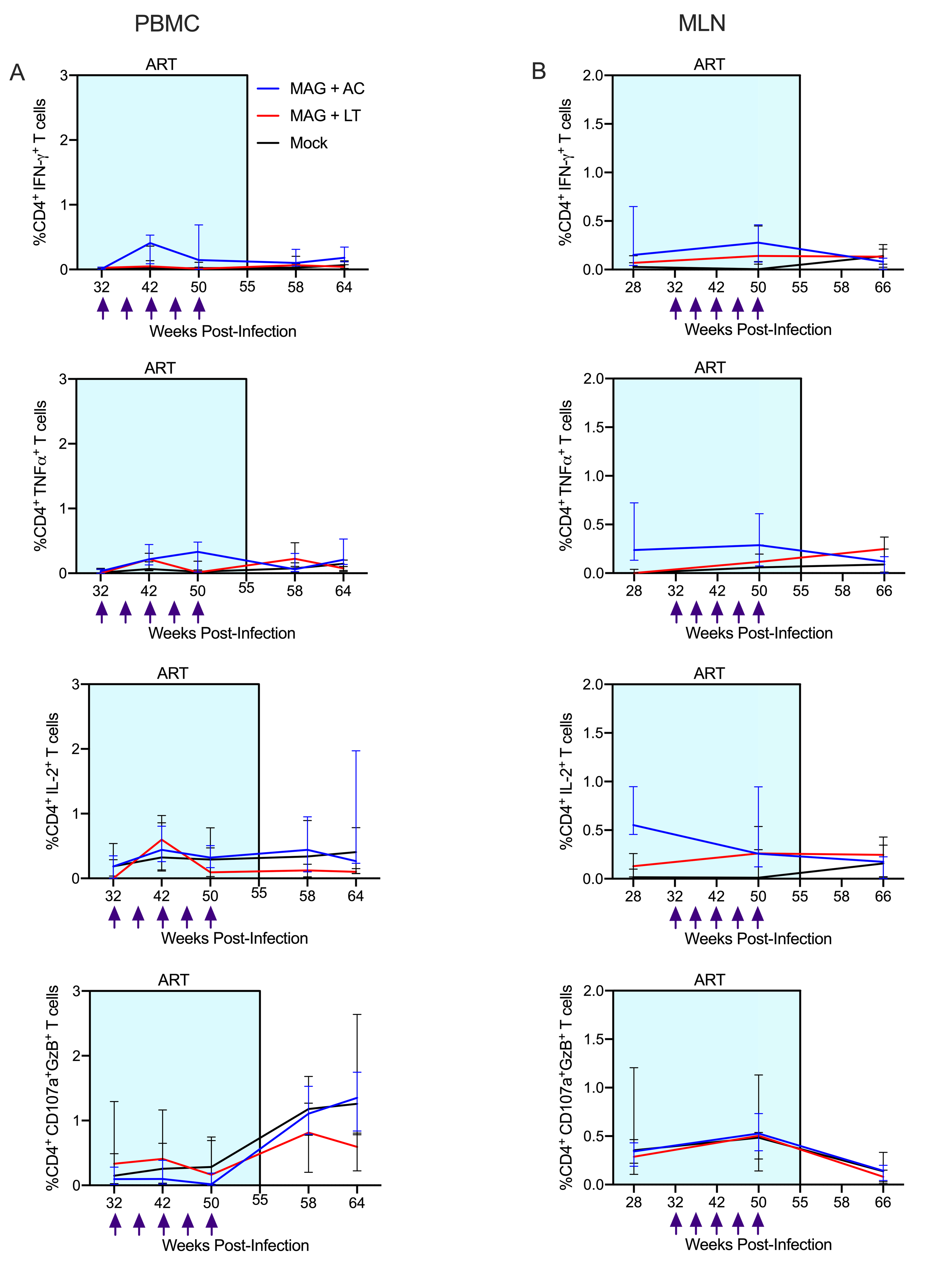

Supplement: S3 Fig — (A) PBMCs were thawed and stimulated with Gag peptides, and expression of IL-2, IFN-γ, TNFα and CD107a/GzB were quantified using intracellular cytokine staining. Shown are the medians and interquartile ranges of each group’s SIV-Gag specific T cell response. (B) Lymphocytes isolated from MLN were thawed and stimulated with Gag peptides, and expression of IL-2, IFN-γ, TNFα and CD107a/GzB were quantified using intracellular cytokine staining. Shown are the medians and interquartile ranges of each group’s SIV-Gag specific T cell response. (A, B) Statistics. Statistical comparisons between baseline and post-vaccination timepoints within a group were calculated using a Wilcoxon matched-pairs signed rank test. A Dunn’s multiple comparisons test was used when making multiple comparisons between vaccine groups and the mock group. Results are considered significant if P ≤ 0.05. (TIF) [file pone.0253265.s003.tif]

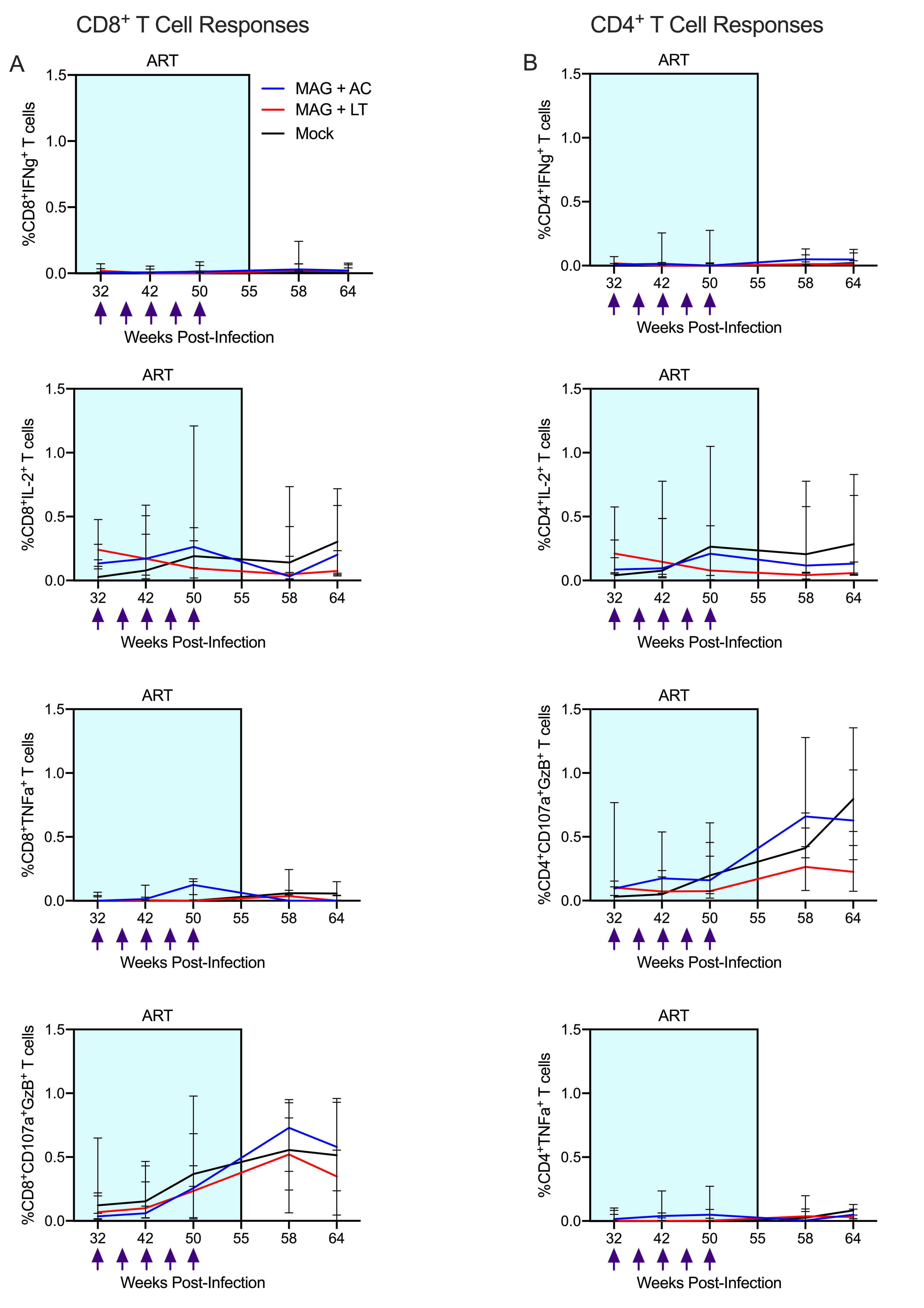

Supplement: S4 Fig — (A-B) PBMCs were thawed and stimulated with Env peptides, and expression of IL-2, IFN-γ, TNFα and CD107a/GzB were quantified using intracellular cytokine staining. Shown are the medians and interquartile ranges of each group’s SIV-Env specific T cell response. (A, B) Statistics. Statistical comparisons between baseline and post-vaccination timepoints within a group were calculated using a Wilcoxon matched-pairs signed rank test. A Dunn’s multiple comparisons test was used when making multiple comparisons between vaccine groups and the mock group. Results are considered significant if P ≤ 0.05. (TIF) [file pone.0253265.s004.tif]

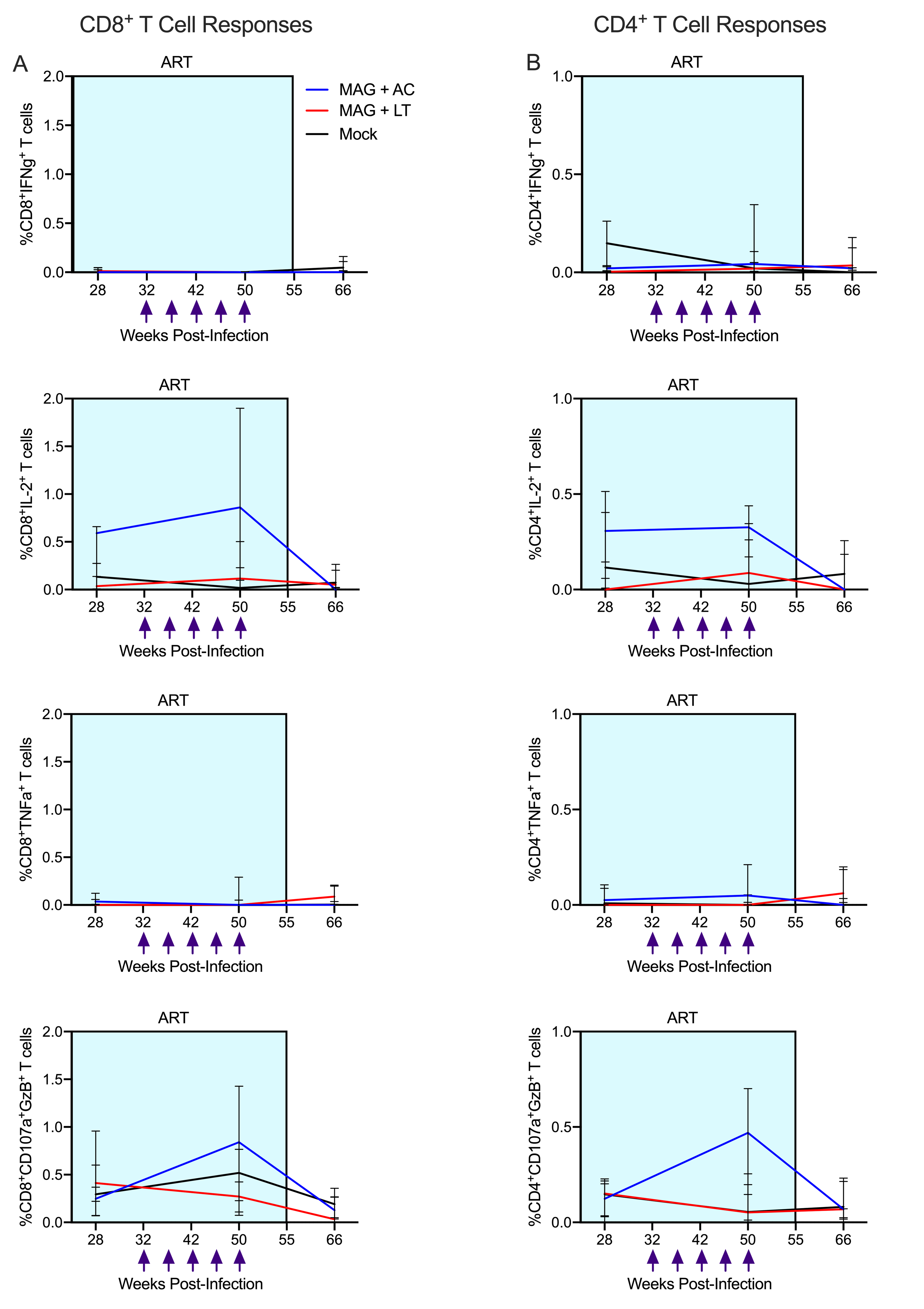

Supplement: S5 Fig — (A-B) Lymphocytes isolated from MLNs were thawed and stimulated with Env peptides, and expression of IL-2, IFN-γ, TNFα and CD107a/GzB were quantified using intracellular cytokine staining. Shown are the medians and interquartile ranges of each group’s SIV-Env specific T cell response. (A, B) Statistics. Statistical comparisons between baseline and post-vaccination timepoints within a group were calculated using a Wilcoxon matched-pairs signed rank test. A Dunn’s multiple comparisons test was used when making multiple comparisons between vaccine groups and the mock group. Results are considered significant if P ≤ 0.05. (TIF) [file pone.0253265.s005.tif]

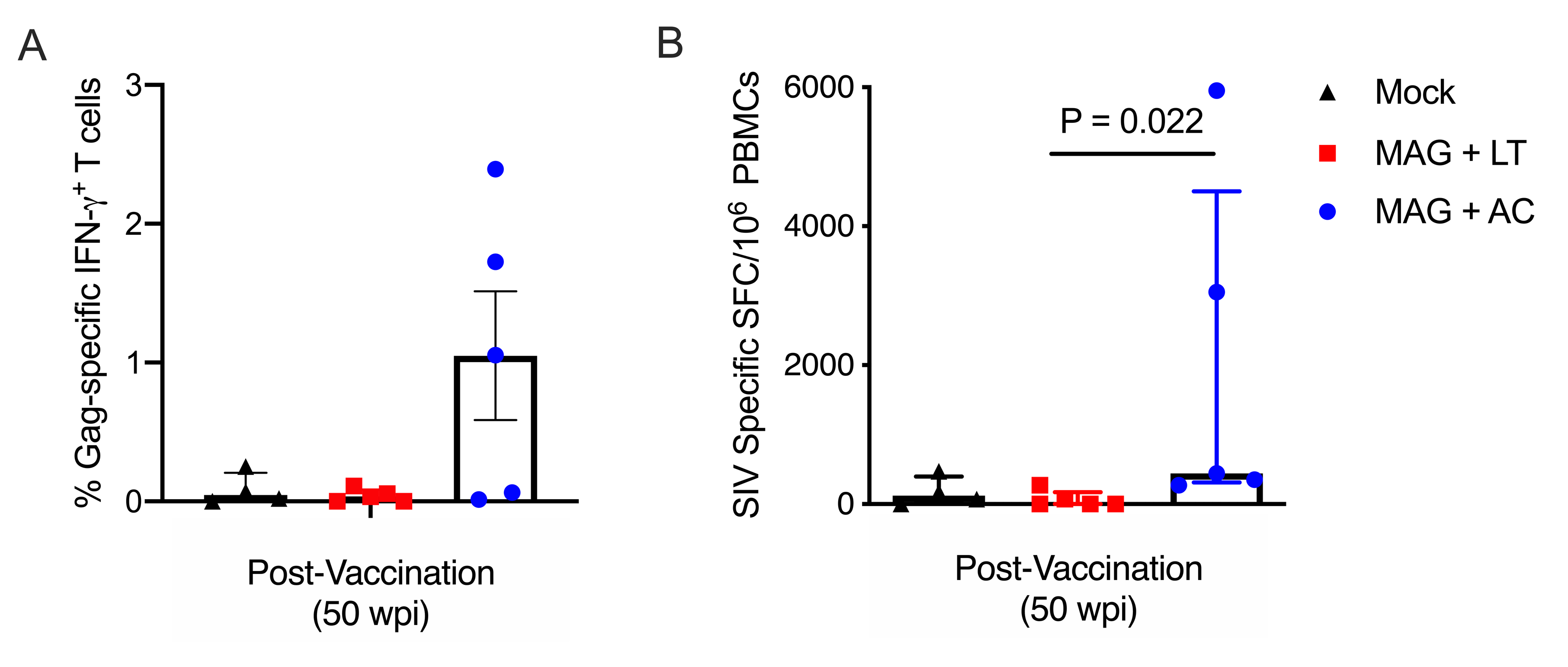

Supplement: S6 Fig — (A) PBMCs were thawed and stimulated with Gag peptides and expression of IFN-γ was quantified using intracellular cytokine staining. Shown are the medians and interquartile ranges of each group’s SIV-Gag specific T cell response. (B) Bulk PBMCs were stimulated with Gag peptides to quantify the SIV-specific IFN-γ response. Results were considered positive if peptide-specific responses were at least twice that of the negative control plus at least 0.01% after background (DMSO) subtraction. Shown are medians and interquartile ranges with data from individual animals layered over each bar. (A-B) A Dunn’s multiple comparisons test was used when making multiple comparisons between vaccine groups and the mock group. Results are considered significant if P ≤ 0.05. (TIF) [file pone.0253265.s006.tif]

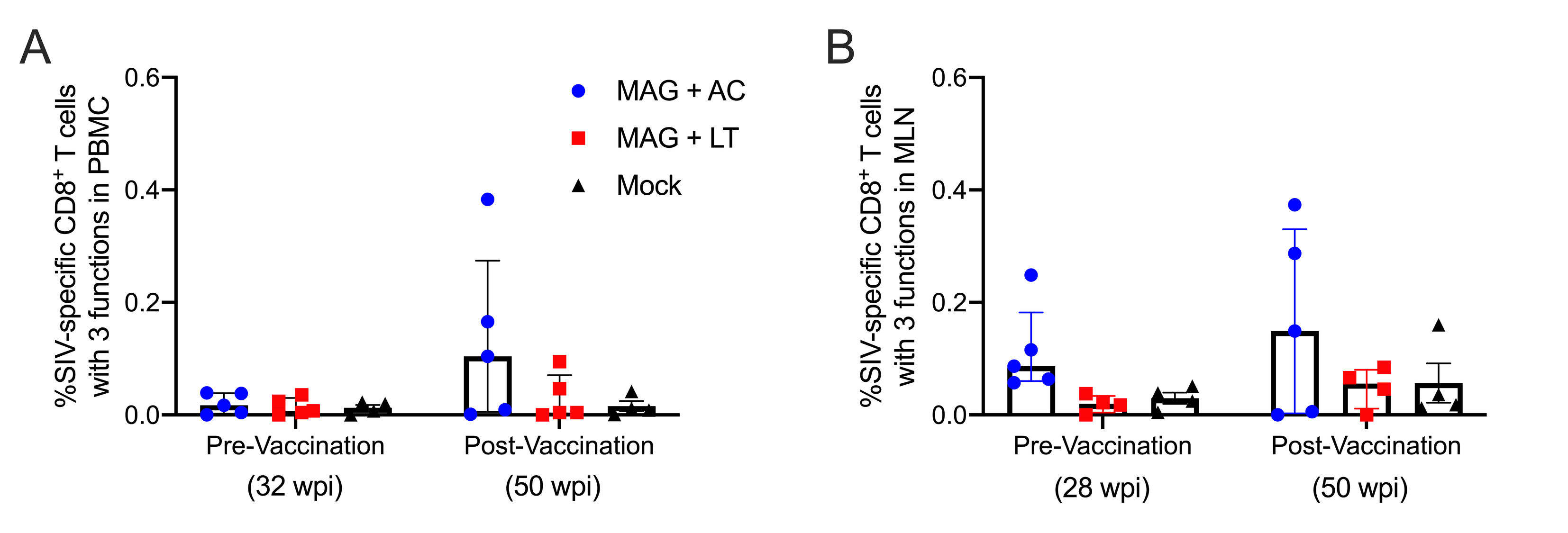

Supplement: S7 Fig — (A-B) PBMCs and lymphocytes from MLNs were thawed and stimulated with Gag and Env peptides, and expression of IL-2, IFN-γ, TNFα and CD107a/GzB were quantified using intracellular cytokine staining. Polyfunctionality is defined as the frequency of T cells specific for Gag or Env and expressing any three or more effector functions. Shown are medians and interquartile ranges with data from individual animals layered over each bar. A Dunn’s multiple comparisons test was used when making comparisons between vaccine groups and the mock group. Results are considered significant if P ≤ 0.05. (TIF) [file pone.0253265.s007.tif]

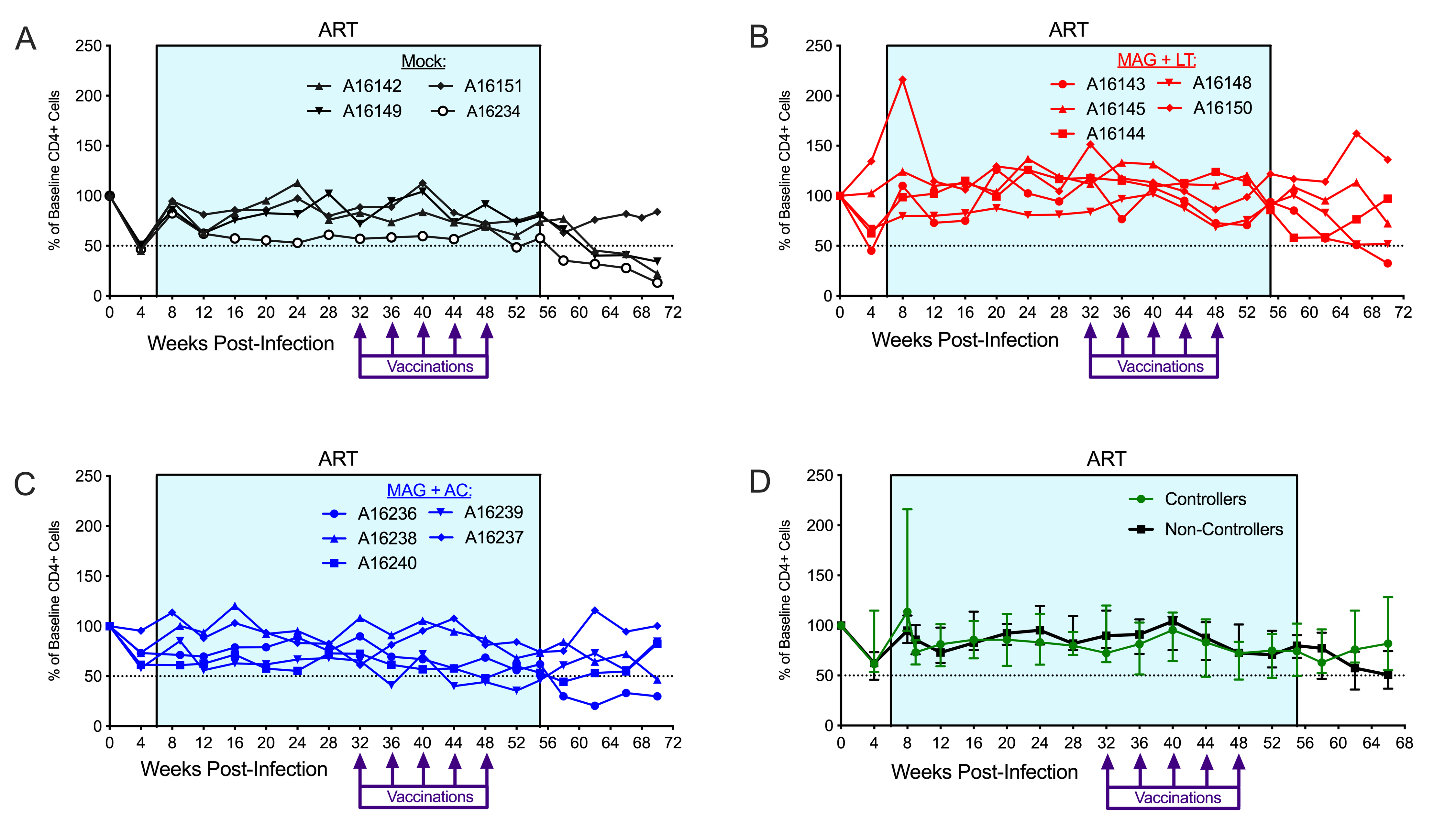

Supplement: S8 Fig — (A-C) Shown are the percent of baseline CD4+ T cell counts for each individual animal in the mock, MAG + LT and MAG + AC groups over time. Percent of baseline CD4+ T cell counts were calculated for the mock, MAG + LT and MAG + AC groups over time by dividing the absolute CD4+ count at a timepoint by the absolute CD4+ count at 0 wpi and multiplying by 100. The dotted line indicates 50% of baseline CD4+ T cells. CD4+ T cell counts were obtained using a Beckman Coulter® AC*T™ 5diff hematology analyzer. (D) Graphed are the median and interquartile range of controllers’ and non-controllers’ percent of baseline CD4+ counts. (TIF) [file pone.0253265.s008.tif]

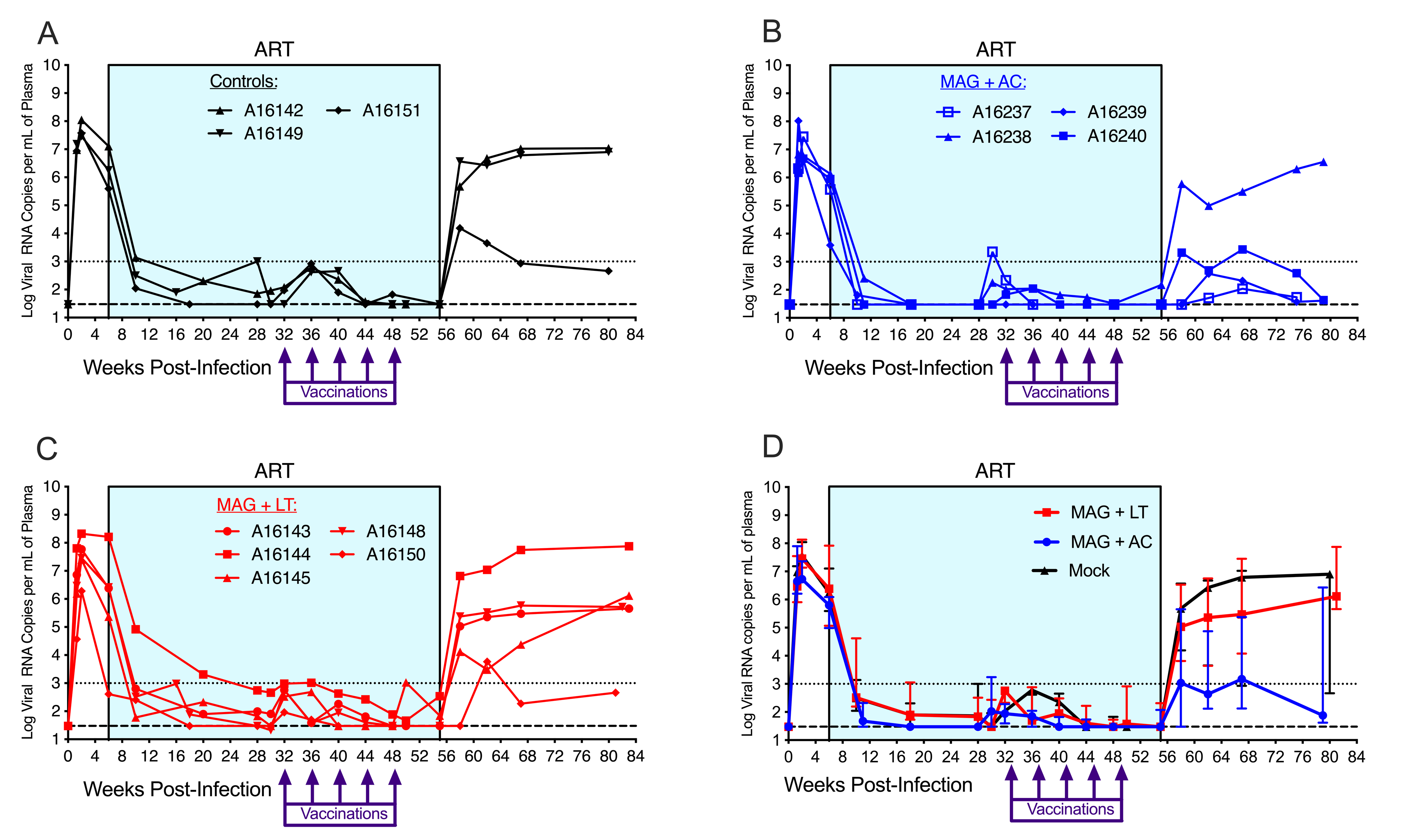

Supplement: S9 Fig — (A-C) Plasma viral RNA was quantified using RT-q-PCR, with a limit of detection of 30 viral RNA copies per 1 mL of plasma, as indicated by the dashed line. ART low responders A16234 and A16236 were removed from the mock-vaccinated group and the MAG + AC group, respectively. (D) Shown are the median viral load and interquartile ranges for each treatment group. The dotted line indicates the threshold for control of virus replication, based on previous studies using SIVΔB670. (TIF) [file pone.0253265.s009.tif]

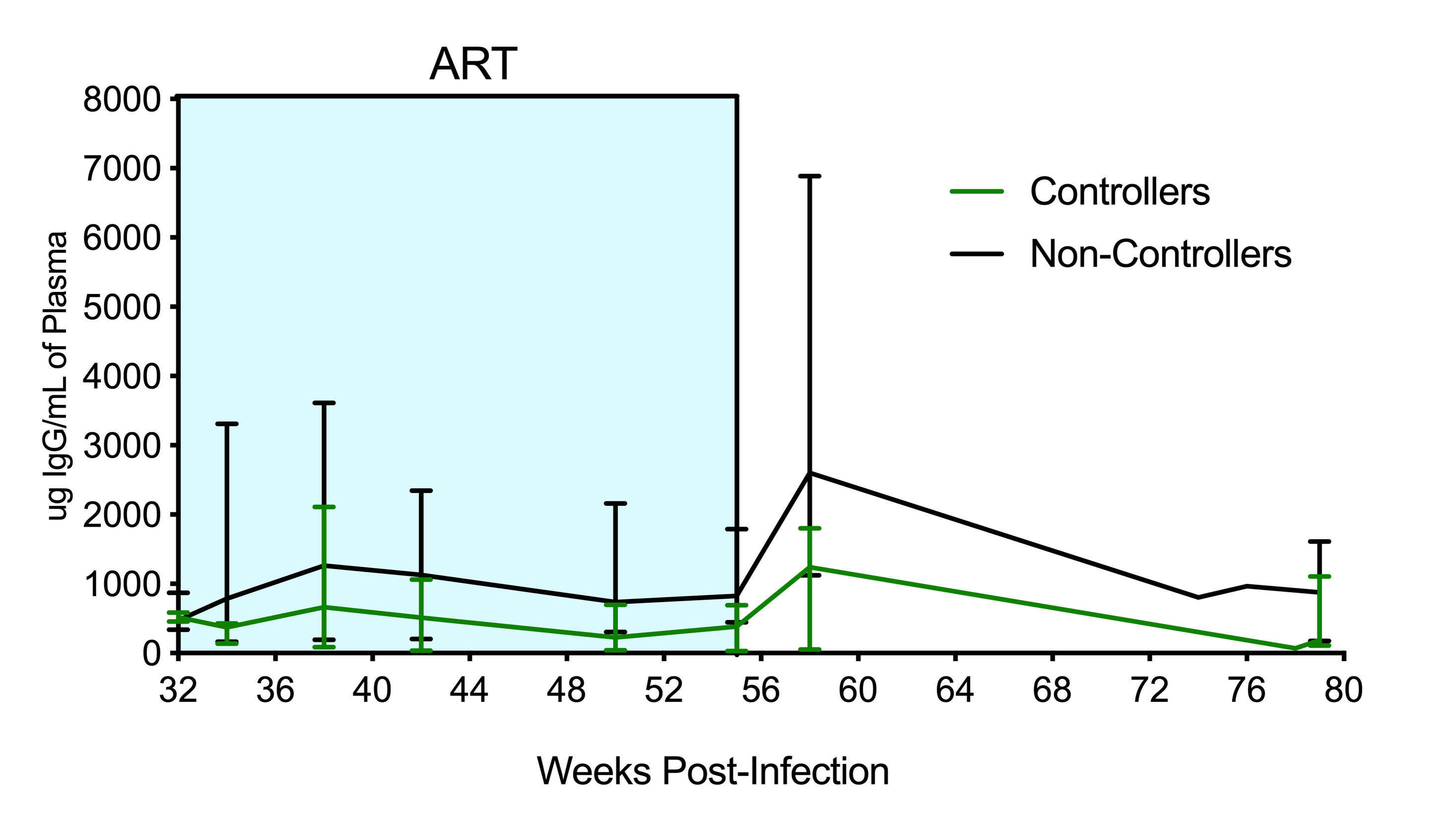

Supplement: S10 Fig — The magnitude of the SIV Env-specific IgG response in the plasma was measured by ELISA, using SIV gp130 as the capture antigen. Shown are medians and interquartile ranges. (TIF) [file pone.0253265.s010.tif]

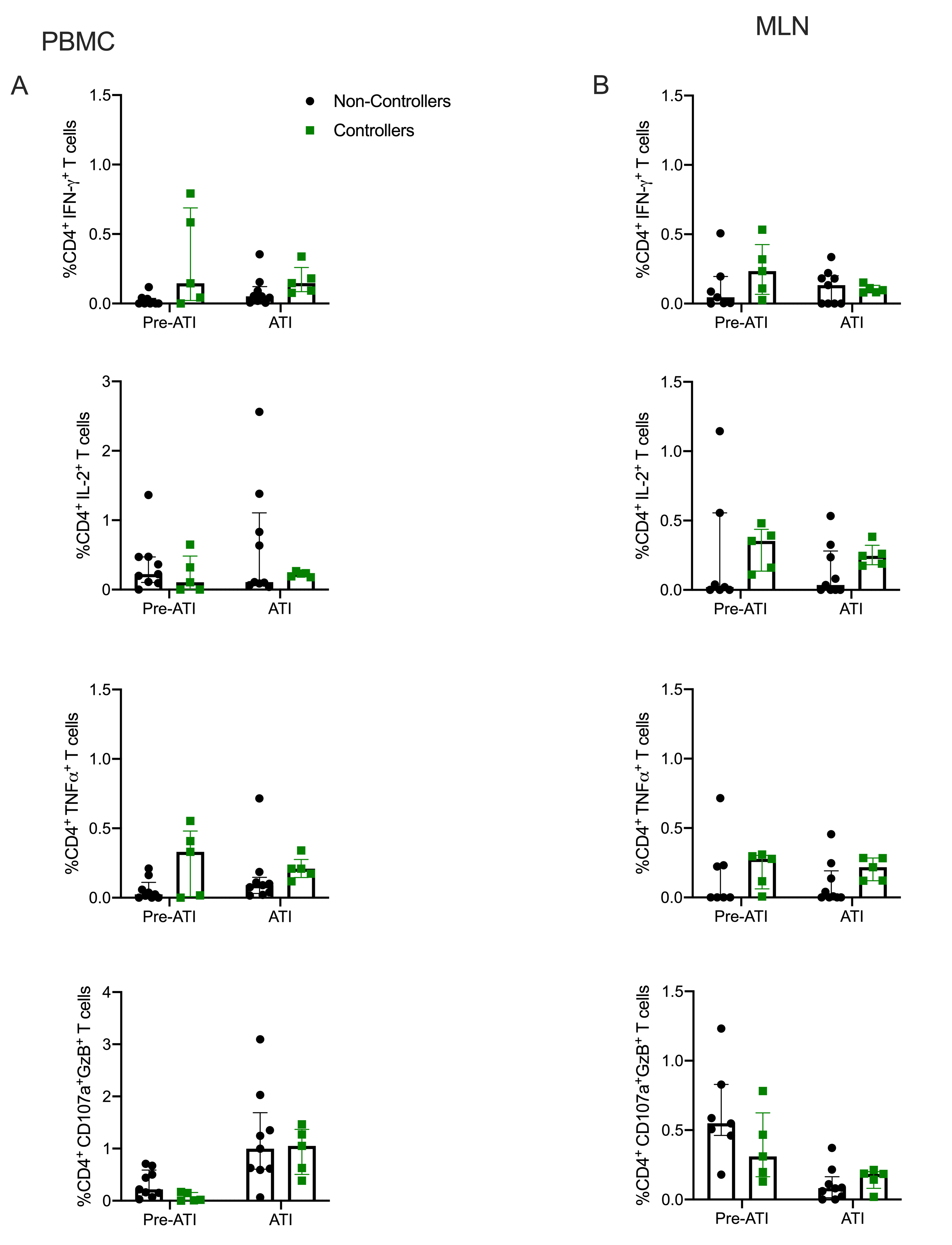

Supplement: S11 Fig — (A-B) PBMCs and MLNs were thawed and stimulated with Gag peptides, and expression of cytokines was quantified using intracellular cytokine staining. Shown are the medians and interquartile ranges of the SIV Gag-specific CD4+ T cell responses of controllers and non-controllers, with individual responses layered over each pre-ATI (50 wpi) and during ATI (62 wpi for PBMC and 66 wpi for MLN). Statistical differences between controllers and non-controllers at each timepoint were assessed using a Mann Whitney t test and the Benjamini-Hochberg method was used to adjust P values. Results are considered significant if P ≤ 0.05. (TIF) [file pone.0253265.s011.tif]

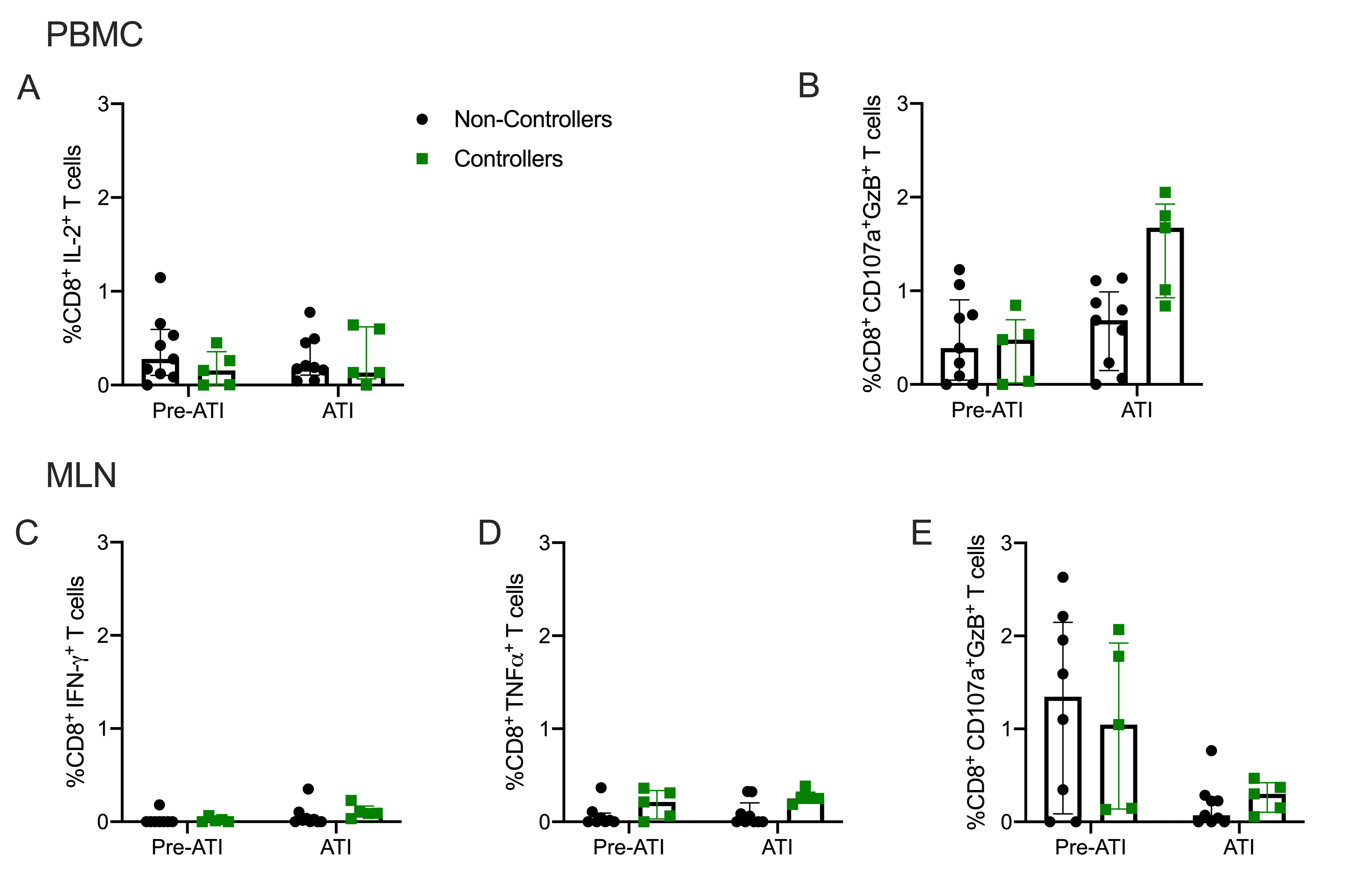

Supplement: S12 Fig — (A-E) PBMCs and MLNs were thawed and stimulated with Gag peptides, expression of cytokines was quantified using intracellular cytokine staining. Shown are the medians and interquartile ranges of the SIV Gag-specific CD8+ T cell responses of controllers and non-controllers, with individual responses layered over each bar pre-ATI (50 wpi) and during ATI (62 wpi for PBMC and 66 wpi for MLN). Statistical differences between controllers and non-controllers at each timepoint were assessed using a Mann Whitney t test and the Benjamini-Hochberg method was used to adjust P values. Results are considered significant if P ≤ 0.05. (TIF) [file pone.0253265.s012.tif]

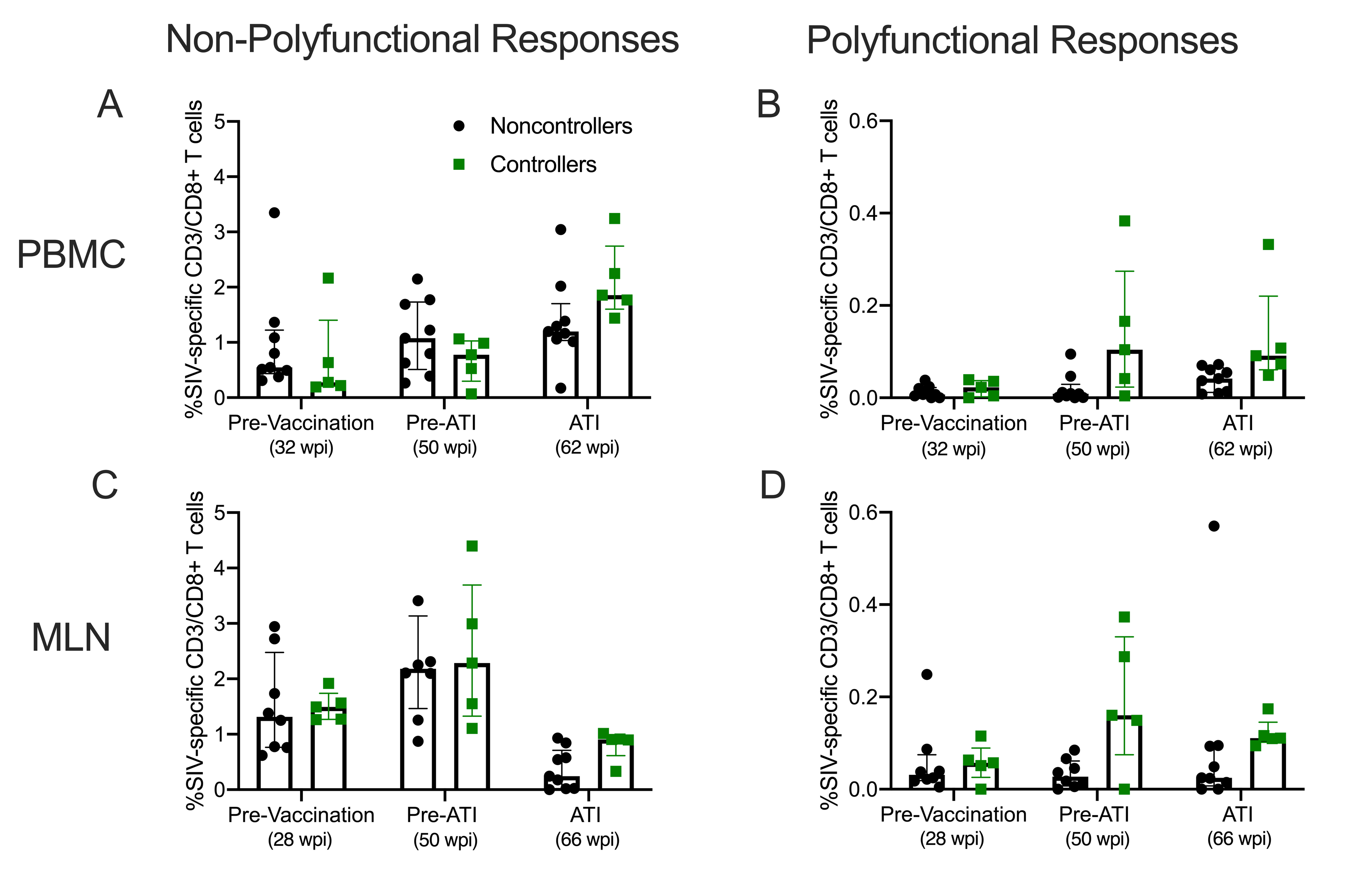

Supplement: S13 Fig — (A-D) PBMCs and lymphocytes from MLNs were thawed and stimulated with Gag and Env peptides, and intracellular cytokine staining was used to quantify expression of IL-2, IFN-γ, TNFα and CD107a/GzB. SIV-specific T cells expressing three or more effector functions are considered polyfunctional, while SIV-specific T cells expressing two or fewer effector functions are considered non-polyfunctional. Shown are medians and interquartile ranges with data from individual animals layered over each bar. At each timepoint, differences between controllers and non-controllers were assessed using a Mann Whitney t test, and the Benjamini-Hochberg method was used to adjust P values. Results are considered significant if P ≤ 0.05. (TIF) [file pone.0253265.s013.tif]

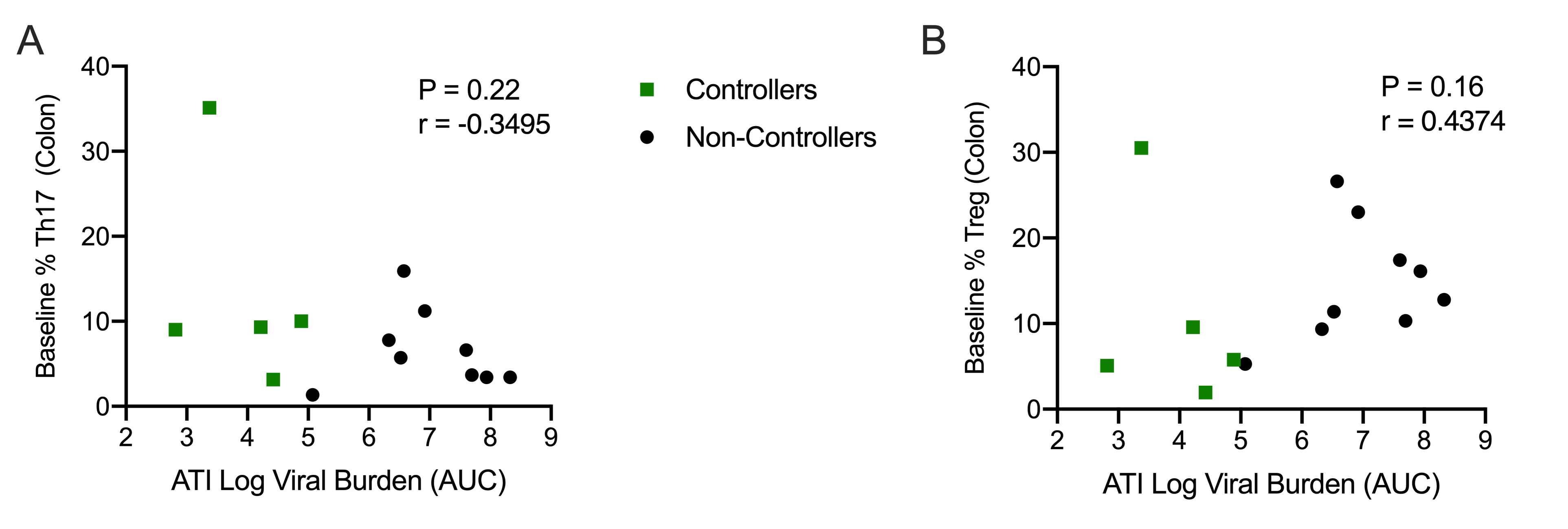

Supplement: S14 Fig — (A-B) Lymphocytes were isolated from colon biopsies and expression of Th17 and Treg markers was quantified using intracellular cytokine staining on fresh cells. A Spearman rank correlation test was used to determine P and r values. Shown are the Benjamini-Hochberg adjusted P values, results are considered significant if P ≤ 0.05. (TIF) [file pone.0253265.s014.tif]
