# Supplementary material for: Effects of therapeutic vaccination on the control of SIV in rhesus macaques with variable responsiveness to antiretroviral drugs
Source: PLoS One. 2021 Jun 17;16(6):e0253265. doi: 10.1371/journal.pone.0253265 (PMC8211199; doi:10.1371/journal.pone.0253265)
Supplement: Attachment — Submitted filename: Response to Reviewers.docx [file pone.0253265.s017.docx]

We thank the reviewers for their continued constructive critique and appreciate the additional opportunity to refine this manuscript. To address each concern, we edited the text as indicated below and added new supplemental data where needed. We believe these revisions fully address the reviewers’ concerns and further improve the focus and impact of the manuscript.

Reviewer 1 Comments:

1) In figure 2F, all IFN-g responses at week 50 in A16236 are against accessory proteins, indicating that MAG+AC immunization did not amplify Gag, Pol, or Env responses. These results should be briefly discussed in the text.

**We agree that this is an important detail that should be addressed. The following text was added (Results, Page 11, Lines 253-256):**

**SIV-specific IFN-γ responses were not observed in one animal in the MAG + LT group (A16150), while one animal in the MAG + AC group (A16236) only exhibited IFN-γ responses to accessory proteins, indicating vaccination had no effect on the SIV-specific IFN-γ response in 1/5 animals in each vaccine group.**

2) Line 484, remove “strong” from this sentence. The data suggest that polyfunctional CD8 T cell responses contribute to controlling virus post-ART but labeling the evidence as “strong” is overstating the data.

**We recognize the importance of presenting our data objectively and removed the word “strong” from the indicated sentence (Page 22, Line 488 after revisions).**

Reviewer 2 Comments:

1) While the authors have addressed most of my concerns, to fully define how intrinsic resistance factors did not play a role in virology outcome, a table showing animal demographics including MHC typing, TRIM5 genotype and viremia status (controller or non-controller) should be included.

**We recognize that certain TRIM5 haplotypes and MHC-1 alleles are associated with improved control of viral replication and can be a confounding factor in vaccine studies. To address this, we included a supplemental table showing each animal’s TRIM5 genotype, virological status, vaccine group, and indicating the presence or absence of MHC-1 alleles associated with lower viral loads. The following text was also edited (Results, Page 14, Lines 317-319):**

**Viral burden during ATI was significantly different between controllers and non-controllers (P = 0.0010, Fig 4B). Certain MHC and TRIM5 genetics have been associated with improved viral control in SIV infected macaques [52-57], however, we observed no association between these MHC or TRIM5 alleles and the control of viral rebound (S1 Table) indicating these genotypes did not likely influence viral burden during ATI in this study.**
